# Choices on contraceptive methods in post-abortion family planning clinic in the northeast Brazil

**DOI:** 10.1186/1742-4755-7-5

**Published:** 2010-05-10

**Authors:** Ana Laura CG Ferreira, Ariani I Souza, Raitza A Lima, Cynthia Braga

**Affiliations:** 1 Instituto de Medicina Integral Prof Fernando Figueira (IMIP) - Research Department - Rua dos Coelhos, 300 Boa Vista 50.070-550, Recife, Brazil

## Abstract

**Background:**

In Brazil, a Ministry of Health report revealed women who underwent an abortion were predominantly in the use of contraceptive methods, but mentioned inconsistent or erroneously contraceptive use. Promoting the use of contraceptive methods to prevent unwanted pregnancies is one of the most effective strategies to reduce abortion rates and maternal morbidity and mortality. Therefore, providing post-abortion family planning services that include structured contraceptive counseling with free and easy access to contraceptive methods can be suitable. So the objective of this study is to determine the acceptance and selection of contraceptive methods followed by a post-abortion family planning counseling.

**Methods:**

A cross-sectional study was carried out from July to October 2008, enrolling 150 low income women to receive post-abortion care at a family planning clinic in a public hospital located in Recife, Brazil. The subjects were invited to take part of the study before receiving hospital leave from five different public maternities. An appointment was made for them at a family planning clinic at IMIP from the 8^th ^to the 15^th ^day after they had undergone an abortion. Every woman received information on contraceptive methods, side effects and fertility. Counseling was individualized and addressed them about feelings, expectations and motivations regarding contraception as well as pregnancy intention.

**Results:**

Of all women enrolled in this study, 97.4% accepted at least one contraceptive method. Most of them (73.4%) had no previous abortion history. Forty of the women who had undergone a previous abortion, 47.5% reported undergoing unsafe abortion. Slightly more than half of the pregnancies (52%) were unwanted. All women had knowledge of the use of condoms, oral contraceptives and injectables. The most chosen method was injectables, followed by oral contraceptives and condoms. Only one woman chose an intrauterine device.

**Conclusion:**

The acceptance rate of post-abortion contraceptive methods was greater and the most chosen method was the best-known one. Implementing a specialized family planning post abortion service may promote an acceptance, regardless of the chosen method. Most important is they *do *receive contraception if they do not wish for an immediate pregnancy.

## Introduction

Annually around 80 million unwanted pregnancies occur worldwide [1], and most of them were due to the non-use or the inconsistent use of contraceptive methods [2].

In developed countries, the contraceptive methods are accessible and most of the women who had undergone an abortion are in the lesser use of consistent contraception methods, such as condoms, coitus interruptus and the rhythm method [3]. In most of the developing countries, unwanted pregnancies are mainly consequence of restricted access to family planning services [4]. In Brazil, a Ministry of Health report based on a 20 year abortion research data revealed women who underwent an abortion were predominantly in the use of contraceptive methods, but mentioned inconsistent or erroneously contraceptive use [5].

A medical, social and economic impact of unwanted pregnancy was significant. Unsafe abortion was one of its consequences, especially in Latin America and the Caribbean Islands where induced abortion is illegal [6]. In Brazil, abortion is only legal in cases as rape or a risk to the mother's life [7]. Therefore, many Brazilian women who decide to end an unwanted pregnancy are obliged to resort to clandestinely. As a consequence, unsafe abortion is a leading cause of maternal mortality in Brazil [8].

Promoting the use of contraceptive methods to prevent unwanted pregnancies is one of the most effective strategies to reduce abortion rates and maternal morbidity and mortality [9,10]. Therefore, providing post-abortion family planning services that include structured contraceptive counseling with free and easy access to all kinds of contraceptive methods can be suitable [9,11]

Contraceptive counseling could result in an increase of method compliance [4,11,12] as well as encouragement and provide emotional support for women to feel more secure and satisfied with the service and motivate the use of family planning methods[12]. Despite the evidences on the effectiveness of family planning services to increase the acceptability of contraceptive methods of women who had recently had an abortion [4,11,13], even though contraceptive counseling remains one of the least inquired components in post-abortion care program. So, the objective of this present study is to describe the acceptance and the choice of contraceptive method in post abortion in the Northeast region of Brazil.

## Methods

A cross-sectional study was carried out among low income post abortion women from July to October 2008. The social workers from five public maternities in the city of Recife, Northeast of Brazil, were asked to invite the women to participate in a post abortion contraceptive counseling survey at the Instituto de Medicina Integral Professor Fernando Figueira (IMIP), a public hospital.

A visit for follow up was arranged by social workers for 186 women with a gynecologist at a family planning clinic at IMIP from the 8^th ^to the 15^th ^day after they had undergone an abortion.

Interviews on socio-economic information, previous knowledge of contraceptive methods and desire to become pregnant were conducted at consultation time. To assess the subject contraception knowledge, the referred question was: *"Which contraceptive methods do you know of?"*

Every woman received information on contraceptive methods, side effects and fertility. Counseling was individualized and addressed them about feelings, expectations and motivations regarding contraception as well as pregnancy intention. The participants received the chosen method with no cost.

Data were used on Epi Info, version 3.3.4 software and an informed consent was obtained from all the subjects. This study was approved by the Internal Review Board Institution.

## Results

Thirty (16.1%) out of 186 recruited women, did not attend the visitation appointment, 2 (1.7%) refused to participate and 4 (2.1%) were excluded from the study due to: uterus malformation (1) and lived in the countryside (3).

Table 1 shows main characteristics of a study population. The women's age ranged from 15 to 45 years, the average age was 25. About half of the women had 4 to 8 years of schooling (44.7%) and had no income (53.3%). Most women had no children (45.3%) and no prior abortion history (73.4%). More than 80% of the study population had a partner and 103 women (68.6%) reported not taking any contraceptive method at the time of conception. Nineteen (47.5%) out of 40 women who had undergone an abortion previously, reported undergoing unsafe abortion. Slightly more than half of the pregnancies (52%) were unwanted.

**Table 1:** Study population characteristics (n = 150)

Characteristics	N	%
Age (years)		
15-19	..21	14.0
20-39	124	82.7
40-45	...5	3.3
Education (years of study)		
≤ 3	1	0.7
4-8	67	44.7
9-11	65	43.3
> 11 ys	17	11.3
Marital status		
With partner	130	86.7
Without partner	20	13.3
Employment status		
Employed	70	46.7
Not Employed	80	53.3
Number of children		
0	68	45.3
1	48	32.0
2	24	16.0
≥ 3	10	6.7
Previous abortion		
0	110	73.4
1	32	21.3
2	6	4.0
≥ 3	2	1.3
Previous unsafe abortion		
Yes	19	47.5
No	21	52.5
Method at conception*		
Nothing	103	68.6
Oral contraceptive	23	15.4
Condom	11	7.4
Coitus interrupts	6	4.0
Injectable	3	2.0
Rhythm method	2	1.3
Sterilization	2	1.3
Intended pregnancies		
Yes	72	48.0
No	78	52.0

* contraceptive method being used in the cycle in which pregnancy occurred

Table 2 describes women's knowledge on contraceptive methods and their choice followed by family planning clinic visit within fifteen days after undergoing an abortion. Knowledge on contraceptive methods was found to be worldwide. All women in this study reported knowing about condoms, oral contraceptives and injectables, while 92.6% had knowledge on the intrauterine device (IUD), 90.7% on sterilization and 90% on coitus interruptus. Vasectomy was mentioned by 88.7% of them, while over 70% reported the knowledge of emergency contraception. Only 30.7% of the interviewed women mentioned about the diaphragm.

**Table 2:** Number and proportion of women for contraceptive method knowledge and choice

	Methods Knowledge		Method choice	
	**N**	**%**	**N**	**%**
Diaphragm	46	30.7		
Rhythm method	112	74.7		
Emergency contraception	118	78.7		
Vasectomy	133	88.7		
Coitus interrupts	135	90.0		
Sterilization	136	90.7		
Intra uterine device	138	92.6	01	0.7
Condom	150	100.0	22	15.1
Oral contraceptive	150	100.0	49	33.6
Injectable	150	100.0	74	50.7
				
Total			146**	100.0

** 4 women did not choose any method

A hundred and fifty women who attended the family planning clinic and received post-abortion contraceptive counseling, 97.4% accepted the use of at least one contraceptive method. Half of the women chose injectables (74), while the second most chosen method was oral contraceptives (33.6%), followed by condoms (15.1%). Only one woman chose IUD.

## Discussion

This study found a high acceptance rate of post-abortion contraceptive methods by the assisted women indicating benefits to offer specialized family planning services to them after abortion.

Only one-fifth of the study population reported a prior history of abortion. This result might be undersized considering that Latin American women, do not have access to safe, legal abortions in their own countries, and this makes it less likely for them to report previous abortions [14]. Whereas this proportion could be different in regions where abortion is legal as shown in an epidemiologic study involving 2780 Chinese women that 35% of them underwent repeated abortions [10] and a randomized controlled trial with 420 Iceland women showed repeated abortions in 35% in the older age group[15].

In a study [16] carried out in Zimbabwe evaluating the provision of post-abortion family planning services, this study included all women with abortion diagnosis. Some women who have had a spontaneous abortion may become pregnant again in a short term and may not accept post-abortion contraception, whereas some others who have undergone an unsafe abortion could be encouraged to avoid unwanted pregnancies. Although the contraceptive priorities and intentions of these women could be different, even though the acceptance and obtainment of the use of contraceptive methods followed by counseling was close to 100%, with agreement of most previous studies [4,9,13,15,17]. Therefore, the high acceptance rate, irrespective to the kind of abortion can be related to the importance given during contraceptive counseling to the interval between one pregnancy to another.

The knowledge on contraceptive methods among the surveyed women was satisfactory. According to the National Survey on Demography in Women and Child Healthcare, 99.9% of the women in the 15-49 year age-group were aware of at least one contraceptive method [18]. Other two Brazilian studies were conducted in the Southeast [19] and in the Northeast of the country [20] and showed that 92.2% and 95.5%, respectively, all the interviewed women reported to know at least one modern contraceptive method. These data show that contraceptive methods awareness is nationwide.

Despite the high knowledge level on contraceptive methods among the studied women, slightly more than half of the pregnancies (52%) were reported as being unwanted, although only one-third of them were using a contraceptive method at the time of conception. When conception occurred, half of them were taking oral contraceptives, in which this might imply to incorrect method use or method failure.

A gap between the level of knowledge on contraceptive methods and a report on the use of contraceptive at the time of conception was observed in the studied women. This finding may have been the result of a "knowledge of a method" variable that was evaluated as "have heard about it", which may not actually reflect to an adequate knowledge to the method in question. A study was carried out in Serbia with University students and they found an association between knowledge of contraceptive methods and contraceptive use [21]. Another Brazilian study found that a large number of women, who reported that they knew about several contraceptive methods, actually knew very little about them. All of the women were asked specific questions about each contraceptive method, and slightly more than half had erroneous concepts of the methods they claimed to know about [22].

As observed by other studies [18-20,22] oral contraceptives, injectables and condoms were mentioned by every woman and the knowledge level among the studied women was considered highly compared to a similar survey carried out in Recife 11 years ago [23]. On the other hand, when knowledge on condom use was compared among women followed by an abortion in the city of Recife in the past 11 years, their knowledge on contraceptive method had increased from 49.6% in 1997 [23] to 100% to this present study. This increase could be due to the important role in the women's health program and prevention campaigns for Sexually Transmitted Diseases/Acquired Immunodeficiency Syndrome (STD/AIDS) that vigorously promote the condom use. The same may have occurred with emergency contraception, which was mentioned by 78.7% of the sample. This percentage is in agreement with a data published by PNDS (2006), but these findings were higher than the ones in an African University of only 51.4% [24].

Despite the availability and provision of all contraceptive methods, only four methods were accepted by the women in this study followed by counseling. The most popular methods were oral contraceptives and injectables, followed by condom and IUD. A high acceptance of injectables may be due to a more fool-proof method and is likely to be easier to use.

The most known methods were also the most chosen ones with the exception of the IUD, despite being the fourth most known method (92.6%) it was chosen by only one woman. This finding is in accordance to the results of a Tanzanian study which has found that none of the woman chose IUD after post-abortion counseling [4]. Therefore, the fact that the women in this study did not accept an IUD may reflect shortcomings in the education and training of the public healthcare providers. Many physicians may not be convinced of the benefits of an IUD in nulliparas, but may be unaware of the indications and contraindications of the method, and may feel unsecure in which candidates would be appropriate to have the device inserted.

A meta-analysis on the effectiveness of post-abortion contraceptive counseling [25] has found no relationship between this intervention and contraceptive practice, suggesting that a more controlled, randomized clinical trial needs to be carried out.

The limitations are related to the study design, which did not allow to investigate causal claims about the impact of counseling and obtaining information beyond the initial acceptance. Therefore, there is no follow up to know whether or not they continued the use of the contraceptive method that they selected and, even to determine if they were able or not to achieve their sexual and reproductive health objective. In addition to these results in this study, it may not represent the actual happenings if a similar counseling was performed in another site or was done by a non specialized provider.

It is important to mention that in our study, 47.5% of the women reported a history of unsafe abortion and 52% had unwanted pregnancy. Thus, the intervention outcome obtained in this study may have been due to the target population that received the intervention was ideal, improving the effectiveness of contraceptive counseling and increasing the acceptance and use of the methods. To choose the ideal target population for the implementation of contraceptive education has been considered important for the positive response[9,12,16].

These data reinforce the need to implement family planning services targeted to women in post-abortion because it is the ideal period of high contraceptive demand among women reducing the risk of unwanted pregnancy and therefore unsafe abortion.

The most important issue is that women *do *receive contraception if they do not wish for an immediate pregnancy. Implementing a high quality contraceptive counseling and training for health professionals could induce women followed by an abortion to accept contraception. Increasing the availability and provision of contraceptive choices and promoting access to contraceptives could reduce the risk of Brazilian women of unwanted pregnancies and potentially unsafe abortion.

## Competing interests

The authors declare that they have no competing interests.

## Authors' contributions

FALCG and SAI conceived and designed the study. All the authors contributed to the data analysis and made substantial comments and contributions to subsequent drafts and approved the final version.
